# A Finite-Difference Based Parallel Solver Algorithm for Online-Monitoring of Resistance Spot Welding

**DOI:** 10.3390/ma15186348

**Published:** 2022-09-13

**Authors:** Tomas Teren, Lars Penter, Christoph Peukert, Steffen Ihlenfeldt

**Affiliations:** Chair of Machine Tools Development and Adaptive Control, Institute of Mechatronic Engineering, Technische Universität Dresden, 01069 Dresden, Germany

**Keywords:** resistance spot welding, finite difference method, real-time simulation, digital twin

## Abstract

Although resistance spot welding (RSW) was invented at the beginning of the last century, the online-monitoring and control of RSW is still a technological challenge and of economic and ecological importance. Process, material and geometry parameters of RSW are stored in the database of the process control system. Prospectively, these accumulated data could serve as the base for data-driven and physics-based models to monitor the spot weld process in real-time. The objective of this paper is to present a finite-difference based parallel solver algorithm to simulate RSW time-efficiently. The Peaceman–Rachford scheme was combined with the Thomas algorithm to compute the electrical–thermal interdependencies of the resistance spot welding process within seconds. Finally, the electric–thermal model is verified by a convergence analysis and parameter study.

## 1. Introduction

Between 7000 and 12,000 spot welds hold a car body together depending on its size [1] and 91.7 million cars were produced in the automotive industry worldwide [2]. One major technological and economical advantage of resistance spot welding over other joining technologies is the ease of integration in automated production lines. Furthermore, it is a lucrative process in technological and organizational terms. The process setup is simple, and the process cycle is, in the order of milliseconds, very short. After stacking one metal sheet on the top of another, pneumatic driven cylinders move electrode caps to clamp the metal sheet stack. Subsequently, the metal sheets are pressed together and a load of up to several hundred MPa is applied (squeeze time). Then, the electrodes are connected to a voltage, and thus an electric current crosses the metal sheets, in which Joule heat initiates weld nugget growth and sheet metal fusion (welding time). At the end, the electrodes rest briefly upon the metal sheets (hold time) before they are moved to the next weld spot (off time) and the process cycle restarts. The most important quality criterion for spot welds is its tensile strength. It can be determined by destructive test methods such as chisel or tensile test. However, after the test the weld is destroyed, and further deployment is impossible. Alternatively, non-destructive test methods can evaluate the weld quality and warrant further use of the assembly after testing. A widespread non-destructive method is ultrasonic testing which aims at detecting the effective contact area size of the weld joint. If this area exceeds a minimal threshold, the joint weld is accepted and dismissed otherwise. The equipment is expensive, requires qualified staff, and ultrasonic testing results scatter broadly. It tends to underestimate the welding spot diameter by approximately up to 2.5 mm [3]. Other non-destructive methods are numerical methods that simulate nugget growth and geometric parameters, such as nugget diameter and penetration depth, which—due to their correlation with weld strength—allow an indirect assessment of the weld joint quality. However, current RSW models are too slow for integration into real-time monitoring and control systems, as discussed in the state-of-the-art section. An online monitoring system for RSW bears the potential of adjusting welding process parameters from one manufactured spot weld to the next in the assembly line, and, as a consequence, reducing the number of NOK welds and save time, costs, and energy. In the early stages, refs [4,5] applied the finite-difference method (FDM) to electro-thermal models of RSW. With the assumption of a constant electric current, ref [4] proposed a model to predict the temperature distribution as a function of time and space, allowing for variations in the mechanical properties of the sheet metal. Ref [5] presents the temperature-dependent electrical potential distribution in the base metal and the interfaces for various electrical currents. In both studies, the nugget diameter and penetration depth derived from the computed temperature field showed good agreement with experimental data. Ref [6] used a control volume formulation and central differences to model the dimensionless temperature field, the nugget growth for different welding currents, electrode tip shapes and thickness ratios of work pieces. The enthalpy-temperature relation was capitalized to account for the phase change. The simulation results in terms of weld nugget growth, nugget thickness and shape were consistent with experimental results. In [7] the finite volume method (FVM) was adopted to build a complex RSW simulation model, which considered—among other aspects—the electric current density, the magnetic field intensity, the temperature, and the velocity field for work pieces with flat faced or truncated electrodes. The effects of the electrode face radius and cone angle on transport mechanism, for example, mass transfer, and various other non-linear phenomena were clearly demonstrated; simulation results agreed well with experimental data. In [8], the mass, momentum, heat and species transport, as well as the magnetic field intensity, were discretized by a control-volume formulation to compute the dynamic electrical resistance during RSW. The simulation result suggest that the dynamic resistance of AISI 1008 steel can be divided into four distinct stages, in which the contact resistance and the bulk resistance contributions vary over time. Several years later, ref [9] developed a control volume based finite difference code for the electrical and thermal field and combined it with a commercial code that provided the mechanical model. Based on this hybrid-approach, the computed nugget size deviated from the experimental data by merely three percent. Many simulations of RSW are based on the finite-element method (FEM), which derives model equations from integration over the finite-element domain. For example, the general-purpose simulation program ABAQUS© (Version 5.7) was used to conduct a parametric study on different electrode shapes, welding currents, and electrode forces for Al-alloys in [1]. Ref [10] analyzes the influence of electrode-water cooling on welding of aluminum alloys AA5182. It utilizes LS-DYNA© (R11.0) to build a thermal-electrical-mechanical model. The simulation results indicated that water cooling affects the temperature distribution in the sheets only slightly, and thus, does not influence nugget growth at all. However, it has a significant effect on the electrode cooling during hold-time. Another study on aluminum alloys for RSW processes was conducted in [11], where a calibrated contact resistance model for AA5182 was presented. The underlying electric–thermal–mechanical FE model could reproduce weld nugget diameters deviating from real experiments by four percent. FEM-based, SORPAS© 3D is a special purpose simulation program with a multi-physics model for RSW [12]. It was applied to investigate short-pulse welding on aluminum alloy AA6016-T4 to reduce the required energy for producing sound welds in thin sheets [13]. The nugget formation was found to happen in two distinct phases: the nucleation, in which 60–80% of the final diameter evolves due to high contact resistance, and the growth stage, when further nugget growth is induced by heat conduction. Savings of approximately 50% regarding energy and time were achieved. SORPAS©.2D provides simulation results with high accuracy, but it requires approximately an hour to run an RSW-simulation with a resolution of 1000 finite elements on a conventional desktop computer [14].

In view of the development labor in past decades, it becomes clear that in the framework of appropriately set model assumptions and on the fundament of suitable material and process data, the nugget growth of RSW can be simulated with sufficient accuracy. Hitherto, numerical analyses of RSW in academia and commercial special purpose programs paid attention to simulation accuracy rather than computational speed; none of the previously cited research papers indicated information on the simulation run-time. In terms of a model-based real-time monitoring and quality assessment system, two essential requirements can be formulated: it should be capable of differentiating between OK and NOK spot welds (sufficient model accuracy) and the time window for the quality assessment is to be shorter than the time between two consecutive spot welds (computation speed). In numerical simulation, these requirements contradict each other, that is, increased simulation accuracy comes along with increased computational time. In the sense of this optimization problem, it is reasonable to constrain the simulation model to physical phenomena, which are predominantly relevant to RSW, to reduce the computation cost. RSW is based on Joule heating, i.e., the transformation of electrical to thermal energy, to join metal stacks by fusion. Thus, the electric-thermal model is considered as the core of any multi-physics model for RSW. The finite difference method, for heat transfer in solids elaborately described in [15], derives model equations from replacing partial derivative terms by finite differences. In this paper, this numerical method is applied to develop an electric-thermal model for RSW by means of the Peaceman–Rachford scheme. It leads to a set of linear equations with tridiagonal band matrices which are solved by the Thomas Algorithm rapidly. Based upon this solving algorithm, the electric-thermal model is verified and investigated on its suitability for real-time simulations.

## 2. Resistance Spot Welding Model

This paragraph describes all aspects necessary for implementing the electric-thermal model presented in this paper. It includes the model geometry, the boundary conditions (Section 2.1), the electric model (Section 2.2), the thermal model (Section 2.3), the material model (Section 2.4), and the solution methodology (Section 2.5).

### 2.1. Model Geometry and Boundary Conditions

According to DIN EN ISO 5821-C0-16-23 the electrode cap geometry was defined. Axial symmetry along the faying surface is supposed and, thus, it suffices to model the upper electrode cap and sheet of the weld joint. Furthermore, the electric-thermal field is assumed to be constant in circumferential direction, which allows the cylindrical coordinate system of the plane (r, z) to represent the cylindrical coordinate system of the three-dimensional space (r, ϕ, z). The modelled plane consists of 1007 nodes and can be considered as the entity of three connected rectangle regions I, II, and III. They are meshed equidistantly and consist of 6 × 20 (I), 19 × 37 (II), and 23 × 8 (III) nodes along the r- and z-axis, respectively. The model geometry as well as its dimensions are depicted in Table 1. To determine the space increment, the criterion for explicit methods Equation (1) is applied. Rearranging it leads to

(1) $$\Delta r=\Delta z\geq \sqrt{2\cdot a_{\max}\cdot \Delta t_{\max}}.$$

In the study at hand, the maximum thermal diffusivity is associated with the copper electrode $a_{\mathrm{Cu}}=\lambda \cdot c_{p}^{-1}\cdot \rho^{-1}=1.01748\times 10^{-4}m^{2}s^{-1}$ and the largest time increment is ${\Delta t}_{\max}=6\times 10^{-4}\;s$. Thus, the space increments are selected as Δr=Δz=0.4 mm.

In order to solve for the temperature and electric potential fields, the boundary conditions of the electric–thermal model must be specified. The electric current streams unidirectional, i.e., a direct current is adopted. Except from the top of the upper electrode, Equation (2), and the faying surface, Equation (3), all system border nodes are electric isolators Equations (4) and (5).

(2) $${\Phi |}_{\mathrm{CD}}=\Phi_{\mathrm{Electrode}}$$

(3) $${\Phi |}_{\mathrm{OG}}=0\;V$$

(4) $${\frac{\partial \Phi }{\partial r}|}_{\mathrm{OA}}={\frac{\partial \Phi }{\partial r}|}_{\mathrm{BC}}={\frac{\partial \Phi }{\partial r}|}_{\mathrm{DE}}={\frac{\partial \Phi }{\partial r}|}_{\mathrm{FG}}=0$$

(5) $${\frac{\partial \Phi }{\partial z}|}_{\mathrm{AB}}{=\frac{\partial \Phi }{\partial z}|}_{\mathrm{EF}}=0$$

The initial temperature of the sheet and electrode, modelled by Equation (6), corresponds to the assumed ambient temperature of 20 °C. Convection between the cooling water and the electrode is modelled as a Dirichlet boundary condition by loading a constant temperature of 20 °C onto separating border nodes, Equation (6). Due to Joule heating, each node acts as a volumetric heat source, and heat is transferred to cooler adjacent nodes. The interfacial heat generation between the sheets is accounted for by Equation (7). Convective heat transfer to the surrounding air is negligible [10,16]. Therefore, the nodes contacting the surrounding air, Equation (8), and the remaining ones, Equation (9), simulate adiabatic system borders.

(6) $${T|}_{t=0}={T|}_{\mathrm{AB}}={T|}_{\mathrm{BC}}=20\;{}^{\circ}C$$

(7) $${Q|}_{\mathrm{OG}}=\frac{1}{\sigma_{\mathrm{contact}}}{\left(\frac{\partial \Phi }{\partial z}\right)}^{2}$$

(8) $${\frac{\partial T}{\partial z}|}_{\mathrm{DE}}={\frac{\partial T}{\partial z}|}_{\mathrm{EF}}=0$$

(9) $${\frac{\partial T}{\partial r}|}_{\mathrm{OA}}={\frac{\partial T}{\partial r}|}_{\mathrm{CD}}={\frac{\partial T}{\partial r}|}_{\mathrm{FG}}={\frac{\partial T}{\partial r}|}_{\mathrm{OG}}=0$$

Material properties of Cu are assigned to the electrode–sheet interface. The estimation of heat transfer across the interfaces is uncertain. Hence it is simplified and treated as heat conduction in a solid body.

### 2.2. Electric Model

Equation (10) is a second order partial differential equation of elliptic type and the model equation for the electrical field. It is solved for the electrical potential, which is used to calculate the Joule heating in Equation (14).

(10) $$\frac{\partial }{\partial r}\left(\frac{1}{\sigma }\frac{\partial \Phi }{\partial r}\right)+\frac{1}{\sigma r}\left(\frac{\partial \Phi }{\partial r}\right)+\frac{\partial }{\partial z}\left(\frac{1}{\sigma }\frac{\partial \Phi }{\partial z}\right)=0.$$

Finite difference methods discretize partial differential equations by replacing derivatives with finite differences, which are obtained by a Taylor series approximation. Equation (10) factors in resistance as function of the space coordinates (r, z) and the temperature T. Applying the finite difference method to Equation (10) yields the finite-difference form of the partial derivatives:

(11) $$\frac{\partial }{\partial r}\left(\frac{1}{\sigma }\frac{\partial \Phi }{\partial r}\right)\approx \frac{1}{{\Delta r}^{2}}\left(\frac{\Phi_{i-1,j}-\Phi_{i,j}}{\sigma_{i-1,j}+\sigma_{i,j}}-\frac{\Phi_{i,j}-\Phi_{i+1,j}}{\sigma_{i,j}+\sigma_{i+1,j}}\right),$$

(12) $$\frac{1}{\sigma r}\left(\frac{\partial \Phi }{\partial r}\right)\approx \frac{1}{2\left(i-1\right){\Delta r}^{2}}\left(\frac{\Phi_{i-1,j}-\Phi_{i,j}}{\sigma_{i-1,j}+\sigma_{i,j}}+\frac{\Phi_{i,j}-\Phi_{i+1,j}}{\sigma_{i,j}+\sigma_{i+1,j}}\right),$$

(13) $$\frac{\partial }{\partial z}\left(\frac{1}{\sigma }\frac{\partial \Phi }{\partial z}\right)\approx \frac{1}{{\Delta z}^{2}}\left(\frac{\Phi_{i,j-1}-\Phi_{i,j}}{\sigma_{i,j-1}+\sigma_{i,j}}-\frac{\Phi_{i,j}-\Phi_{i,j+1}}{\sigma_{i,j}+\sigma_{i,j+1}}\right).$$

Substituting the partial derivatives in Equation (10) with Equations (11)–(13) leads to the finite difference model equation, which is used to compute the potential distribution in the electrode-sheet configuration.

### 2.3. Thermal Model

#### 2.3.1. Heat Diffusion Equation

The heat diffusion equation is a second order partial differential equation of parabolic type. For a solid or motionless fluid volume unit, it states that the rate of change of thermal energy stored equals the net rate of in- and outgoing conductive energy transfer and the rate of thermal energy generation. The partial differential equation of the heat diffusion is defined by:

(14) $$\rho \cdot c_{p}\cdot \frac{\partial T}{\partial t}=\frac{\partial }{\partial r}\left(\lambda \frac{\partial T}{\partial r}\right)+\frac{\lambda }{r}\frac{\partial T}{\partial r}+\frac{\partial }{\partial z}\left(\lambda \frac{\partial T}{\partial z}\right)+Q.$$

After discretizing (14) by the finite difference method, the terms are as follows:

(15) $$\frac{\partial }{\partial r}\left(\lambda \frac{\partial T}{\partial r}\right)\approx \left(\frac{\lambda_{i-1,j}+\lambda_{i,j}}{2\Delta r^{2}}T_{i-1,j}-\frac{\lambda_{i+1,j}+2\lambda_{i,j}+\lambda_{i,j}}{2\Delta r^{2}}T_{i,j}+\frac{\lambda_{i+1,j}+\lambda_{i,j}}{2\Delta r^{2}}T_{i+1,j}\right)$$

(16) $$\frac{\lambda }{r}\frac{\partial T}{\partial r}\approx \lambda_{i,j}\left(\frac{T_{i+1,j}-T_{i-1,j}}{2{\Delta r}^{2}\left(i-1\right)}\right)$$

(17) $$\frac{\partial }{\partial z}\left(\lambda \frac{\partial T}{\partial z}\right)\approx \left(\frac{\lambda_{i,j-1}+\lambda_{i,j}}{2\Delta z^{2}}T_{i,j-1}-\frac{\lambda_{i,j+1}+2\lambda_{i,j}+\lambda_{i,j-1}}{2\Delta z^{2}}T_{i,j}+\frac{\lambda_{i,j+1}+\lambda_{i,j}}{2\Delta z^{2}}T_{i,j+1}\right)$$

(18) $$\frac{\partial T}{\partial t}\approx \frac{T^{n+1}-T^{n}}{\Delta t}.$$

Substituting the partial derivative terms in Equation (14) with Equations (15)–(18) leads to the discretized heat diffusion equation, which is applied to compute the temperature field in the electrode-sheet configuration.

#### 2.3.2. Joule Heating

Joule heating connects the electrical potential to the thermal model. It is implemented as the source term in the heat diffusion Equation (14). The formula for Joule heating is defined as Equation (19) and can be discretized by central differences Equations (20) and (21) for the gradient of the electrical potential. After inserting Equations (20) and (21) into Equation (19), the discretized source term emerges and can be embedded in Equation (14).

(19) $$Q=\frac{1}{\sigma }\left({\left(\frac{\partial \Phi }{\partial r}\right)}^{2}+{\left(\frac{\partial \Phi }{\partial z}\right)}^{2}\right)$$

(20) $$\frac{\partial \Phi }{\partial r}\approx \frac{\Phi_{i+1,j}-\Phi_{i-1,j}}{2\Delta r}$$

(21) $$\frac{\partial \Phi }{\partial z}\approx \frac{\Phi_{i,j+1}-\Phi_{i,j-1}}{2\Delta z}$$

#### 2.3.3. Contact Resistance Model

The basics of electrical contacts were studied and published by Holm [17] and Greenwood [18]. The contact resistance can be decomposed into constriction and film resistance. However, the applied equation in this model ignores the distinction and considers both components as one entity for the sake of simplicity. The formula that relates resistance to resistivity is:

(22) $$R=\frac{\sigma \cdot l}{A},$$

where the length l and the area A are the size of a three-dimensional electrical resistor. The model of linear variation of voltage within the contact zone is adopted from [5]. Hence, the electric current density Equation (23), and the interfacial heat generation at the faying surface Equation (24) can be computed by

(23) $$J_{\mathrm{contact}}=\frac{1}{\sigma_{\mathrm{contact}}}{\left(\frac{\partial \Phi }{\partial z}\right)}_{\mathrm{contact}},$$

(24) $$Q_{\mathrm{contact}}=\frac{1}{\sigma_{\mathrm{contact}}}{\left(\frac{\partial \Phi }{\partial z}\right)}_{\mathrm{contact}}^{2}.$$

Equations (23) and (24) indicate that the electric current flows perpendicular through the faying surface. The voltage drop at the faying surface is discretized by the forward difference according to:

(25) $$\frac{\partial \phi }{\partial z}\approx \frac{\phi_{\mathrm{contact},j\;+1}-\Phi_{\mathrm{contact},j}}{{\Delta z}_{\mathrm{contact}}}.$$

The contact layer height between the sheets is set to $\Delta z_{\mathrm{contact}}$= 0.01 mm and is regarded as the average roughness of the faying surface, which in practice deforms more the heavier the electrode load is. According to the literature it ranges between 0.01 and 0.05 mm [5,19].

#### 2.3.4. Phase Change Model

The phase change from solid to liquid is of particular importance. Neglecting the effect would lead to an unrealistically high temperature field beyond the melting point. While the sheet melts, the temperature remains constant and energy—the specific latent heat H—is absorbed to break down the lattice structure of the solid elementary cells. The specific latent heat for Al amounts to 397 kJ/kg. The specific heat capacity of the phase change $c_{\mathrm{phase}}=1.14\;\mathrm{kJ}/\left(\mathrm{kg}\cdot {}^{\circ}C\right)$ results from the arithmetic mean of the specific heat capacity at the solidus and liquidus temperature. For RSW simulations, the latent heat can be transformed into an equivalent temperature difference

(26) $$\Delta T=\frac{H}{c_{\mathrm{phase}}}.$$

It can be considered as an artificial temperature reservoir, which can be used to differentiate between solid, solid–liquid and liquid phase. In the solid state $\left(T_{\mathrm{sheet}}<T_{\mathrm{solidus}}\right)$, ΔT=348.25 °C remains constant; the sheet temperature increases. As the sheet enters the solid–liquid phase $\left(T_{\mathrm{liquidus}}>T_{\mathrm{sheet}}>T_{\mathrm{solidus}}\right)$, the difference temperature $T_{\mathrm{sheet}}-T_{\mathrm{solidus}}$ is subtracted from ΔT. As long as the sheet remains in the solid–liquid phase (0<ΔT<348.25 °C), the temperature increase is suppressed. When ΔT=0 °C is reached, the liquid phase $(T_{\mathrm{sheet}}>T_{\mathrm{liquidus}}$) begins and the model continues to increase the temperature.

### 2.4. Material Model

The material data set encompasses specific heat capacity $c_{p}$, thermal conductivity λ, electric resistance σ and density ρ, all as functions of temperature. The authors in [20] provide data on specific heat capacity for Al and Cu in all three phases; on electric resistance only for phase change and liquid stage. These electrical resistance values were inserted into the Wiedemann–Franz Law Equation (27) to obtain the thermal conductivity.

(27) $$\lambda =\frac{T}{\sigma }\cdot L$$

For solid state, ref [21] provides values for the thermal conductivity of Al and Cu. These served the calculation of electrical resistance by the Wiedmann–Franz Law. The density for phase change and liquid state stem from [20] as well. The solid density values for Cu and Al were determined by

(28) $$\rho \left(T,\alpha \right)=\frac{\rho_{25}}{{\left(1+\alpha \left(T-25\;{}^{\circ}C\right)\right)}^{3}}.$$

The temperature dependent thermal expansion factor α, the density value of the sheet $\rho_{25}^{\mathrm{Al}}=2700\;\mathrm{kg}/m^{3}$ and the electrode $\rho_{25}^{\mathrm{Cu}}=8960\;\mathrm{kg}/m^{3}$ are given in [22]. Depending on temperature intervals, the material data were averaged and are summarized in Table 2.

The solidus temperature for Al is defined at 660 °C. In the simulation, the temperature of the electrode cap never came near solidus temperature. Therefore, material parameters of copper were restricted to solid state exclusively. For each phase a contact resistance was defined. The contact resistances for the solid and solid–liquid phase were aligned with the bulk material resistance of the aluminum sheet as the faying surface and the bulk material are assumed to possess the same consistency beyond solidus temperature.

### 2.5. Solution Methodology

D.W. Peaceman and H. H. Rachford introduced an alternating-direction implicit scheme for finite difference methods—the so-called Peaceman–Rachford scheme [23]. It originates a set of linear equations with tridiagonal band matrices which can be solved by the Thomas Algorithm efficiently. To the author’s best knowledge, this solution methodology was used to simulate the melting during the RSW for the first time. By using the discretized heat diffusion Equation (14), the application of the Peaceman–Rachford scheme is demonstrated in this section. The approach is analogously viable to the discretized form of Equation (10).

#### 2.5.1. Peaceman–Rachford Scheme

The Peaceman–Rachford scheme is an unconditionally stable method permitting an arbitrary large time step size. Locally, it is second order accurate in space and time $O\left({\Delta r}^{2},{\Delta z}^{2},{\Delta t}^{2}\right)$. The heat diffusion equations casted into the Peaceman–Rachford scheme leads to following equations:

(29) $$\left(1-\frac{\mu_{r}}{2}(\delta_{r}^{2}+\frac{\delta_{r}}{\left(i-1\right)})\right)T_{i,j,n+0.5}=(1+\frac{\mu_{z}}{2}\delta_{z}^{2})T_{i,j,n}+\frac{\Delta t}{2}Q_{i,j,n+0.5}$$

(30) $$(1-\frac{\mu_{z}}{2}\delta_{z}^{2})T_{i,j,n+1}=\left(1+\frac{\mu_{r}}{2}(\delta_{r}^{2}+\frac{\delta_{r}}{\left(i-1\right)})\right)T_{i,j,n+0.5}+\frac{\Delta t}{2}Q_{i,j,n+0.5}.$$

The alternating-direction character of the PR-Scheme is clarified by Equations (29) and (30). At first, the known temperature distribution $T_{i,j,n}$ is used to compute the intermediate temperature distribution $T_{i,j,n+0.5}$ in radial direction by Equation (29). Afterwards, this intermediate solution serves as the input for the subsequent calculation carried out by Equation (30), which outputs the temperature distribution $T_{i,j,n\;+1}$ in axial direction. The solution of Equation (30) constitutes the solved temperature field of the resistance spot weld nugget. The difference operators $\delta_{r}T_{i,j},\delta_{r}^{2}T_{i,j},{\mathrm{and}\;\delta }_{z}^{2}T_{i,j}$ are defined as follows:

(31) $$\delta_{r}T_{i,j}=\lambda_{i,j}\frac{T_{i+1,j}-T_{i-1,j}}{2}$$

(32) $$\delta_{r}^{2}T_{i,j}=\frac{\lambda_{i-1,j}+\lambda_{i,j}}{2}T_{i-1,j}-\frac{\lambda_{i+1,j}+2\lambda_{i,j}+\lambda_{i-1,j}}{2}T_{i,j}+\frac{\lambda_{i+1,j}+\lambda_{i,j}}{2}T_{i+1,j}$$

(33) $$\delta_{z}^{2}T_{i,j}=\frac{\lambda_{i,j-1}+\lambda_{i,j}}{2}T_{i,j-1}-\frac{\lambda_{i,j+1}+2\lambda_{i,j}+\lambda_{i,j+1}}{2}T_{i,j}+\frac{\lambda_{i,j+1}+\lambda_{i,j}}{2}T_{i,j+1}.$$

The Equations (29) and (30) must be arranged according to $a_{i}T_{i-1,j,n+0.5}+b_{i}T_{i,j,n+0.5}+c_{i}T_{i+1,j,n+0.5}=d_{i,n}$ and $a_{j}T_{i,j-1,n+1}+b_{j}T_{i,j,n+1}+c_{j}T_{i,j+1,n+1}=d_{j,n+0.5}$, respectively, in order to be formatted appropriately for the application of the Thomas-Algorithm.

#### 2.5.2. Thomas Algorithm and Code Implementation

The Peaceman–Rachford scheme leads to a system of algebraic equations, one for each of Equations (29) and (30). The Thomas algorithm as a direct solver for tridiagonal system of algebraic equations, treats Equations (29) and (30) indifferently, i.e., it is applied to both matrix equations equally. Thus Equations (29) and (30) can be unified in a general format:

(34) $$\left[\begin{array}{cccccccc} & b_{1} & c_{1} & & & & & \\ & a_{2} & b_{2} & c_{2} & & & & \\ & & a_{3} & b_{3} & c_{3} & & & \\ & & & \ddots & \ddots & \ddots & & \\ & & & & a_{N-1} & b_{N-1} & c_{N-1} & \\ & & & & & a_{N} & b_{N} & \end{array}\right]\left[\begin{array}{c} T_{1}\\ T_{2}\\ T_{3}\\ \vdots \\ T_{N-1}\\ T_{N} \end{array}\right]=\left[\begin{array}{c} d_{1}\\ d_{2}\\ d_{3}\\ \vdots \\ d_{N-1}\\ d_{N} \end{array}\right].$$

The Thomas Algorithm consists of two phases. First, the matrix equation is brought into an upper diagonal shape by zeroing $a_{k}$, and substituting $b_{k}$ and $d_{k}$ by

(35) $$b_{k}=b_{k}-\frac{a_{k}}{b_{k-1}}c_{k-1}$$

(36) $$d_{k}=d_{k}-\frac{a_{k}}{b_{k-1}}d_{k-1}.$$

for k=2,…,N. Second, the temperature field is solved by backward substitution, based on

(37) $$T_{k}=\frac{d_{k}-c_{k}T_{k+1}}{b_{k}},$$

beginning in the last row with $T_{N}=d_{N}/b_{N}$ towards the first row with $T_{1}$. To provide an overview of the coupling between the electric and the thermal model and the underlying program structure, a pseudo-code is depicted in the Appendix A. After initialization of the model geometry and fixed material properties, the welding time $t_{w}$ can be defined as the product of the time step Δt and the number of time steps n arbitrarily. The number of time steps n also determines how often the temperature dependent material properties are updated as well as how many times the electric and thermal fields are calculated. The electrical field is computed iteratively until the residuum and the difference of succeeding solutions fall below given predetermined break conditions, respectively. By combining the Peaceman–Rachford Scheme with the Thomas algorithm, the temperature field is calculated by two main sequences, which are referred to as sweeps or scans in pertinent literature. During the first sweep, intermediate temperature values are calculated for each row grid point wise from left to right by Equation (29). Analogously to the first sweep, the second sweep calculates the final temperature values for each column grid point wise from bottom to top by Equation (30). For further details on the theory and implementation of the Peaceman–Rachford scheme and the Thomas Algorithm, the books [24,25] can be consulted. This solver methodology operates line by line, which makes parallel computing feasible and promotes real-time simulation. The simulation model was run on an Intel Core i5-6500 CPU (3.2–3.6 GHz) and in MATLAB© (R2018b).

## 3. Results

Paragraphs 3 and 4 aim at verifying the implemented model from a numerical and physical point of view by a convergence analysis and a parameter study, respectively. It demonstrates that the electric-thermal model meets general expectations on the behavior of numerical methods and on the physics of RSW. Despite the Joule heating in the electrode has also been part of the computation and influenced the simulation run-time, it is not analyzed in detail in the forthcoming paragraphs due to its negligible low temperature increase.

### 3.1. Convergence Analysis and Computation Speed

In order to examine the electro–thermal model’s run time $t_{\mathrm{sim}}$ and convergence behavior, three spot welds with welding times $t_{w}=40\;\mathrm{ms}$, $t_{w}=50\;\mathrm{ms}$ und $t_{w}=60\;\mathrm{ms}$ were simulated. The voltage between the electrode cap and the faying surface drops by 0.5 V and the contact resistance between the sheets amounts to 400 μΩm for all variants. All other simulation parameters are known from preceding paragraphs and are identical for all simulation variants as well. For each welding time $t_{w}=n\cdot \Delta t$, the number of simulations runs n and the time steps Δt were combined twelve times, see Table 3, Table 4 and Table 5. The number of variants per simulated welding time was chosen to be twelve so as to ensure that the mean sheet temperature $\bar{T}$ remains constant when the time step Δt is further decreased (or simulations run n is further increased). Therefore, the twelfth variant of each Table 3, Table 4 and Table 5 is the closest approximation of the assumed exact solution for the corresponding welding time and set spatial grid. The discretization errors ϵ are calculated by referring to the mean temperature $\bar{T}$ of the twelfth variants in all three tables, i.e., $\varepsilon =\left(\bar{T}-{\bar{T}}_{\mathrm{variant}12}\right)/{\bar{T}}_{\mathrm{variant}12}$. The influence of the simulations runs n and the influence of the time step Δt on the convergence behavior were to be analyzed separately. Thus, across the Table 3, Table 4 and Table 5 the simulation runs n were held constant with varying time steps Δt for the variants 1, 2, 4, 5, 7, 8, 10, and 11; while the simulations runs n varied with constant time steps Δt for the variants 3, 6, 9, and 12. The arithmetic mean of the sheet temperature $\bar{T}$ over all nodes and its standard deviation SD($\bar{T}$) as well as the minimum and maximum values of the sheet temperature $T_{\min}$ and $T_{\max}$ were determined. Finally, the run-time $t_{\mathrm{sim}}$ of each simulation variant was measured manually with a stopwatch and is therefore subject to slight measurement errors.

### 3.2. Parameter Study

**Figure 1:**
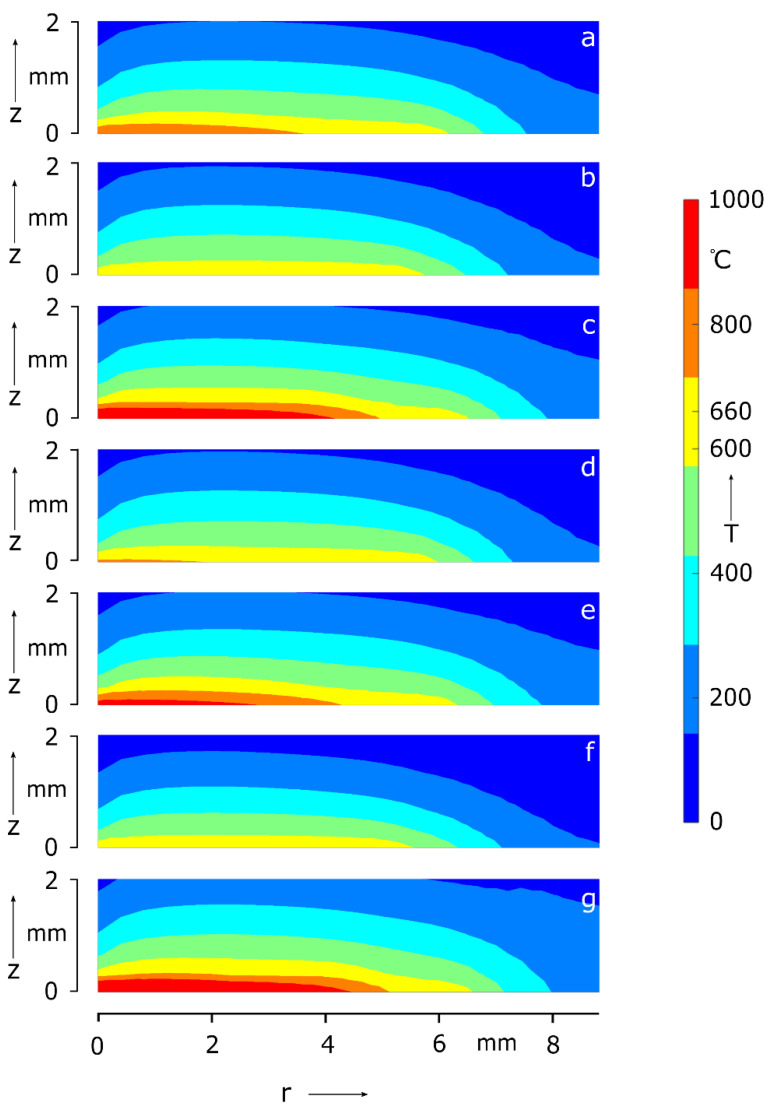
reference spot weld (**a**) and spot welds for varying voltage (**b**,**c**), contact resistance (**d**,**e**) and welding time (**f**,**g**).

For qualitatively verifying the simulation model, n = 10,000, as this choice leads to results with acceptable low discretization error (ϵ ≤ 1%). As process parameters, the welding time, the applied voltage, and the electric current affect the amount of thermal energy produced in the metal sheets. Aside from above mentioned process parameter, the electric contact resistance at the faying surface also influences the Joule heating. Thus, the parameter study is performed by varying the electrode voltage, the welding time and the contact resistance one by one while all other parameters are held constant. The results of the parameter study are shown in Figure 1 with corresponding data in Table 6. The reference spot weld is depicted separately in the first row of Table 6 and referenced by Figure 1a. 

According to Section 2.3.4, a grid point is solid (T < 660 °C), mushy (T = 660 °C) or liquid (T > 660 °C). Thus, the upper limit of the temperature scale in Figure 1 is set to 1000 °C to permit the distinction between these three phases. The simulation of the reference weld (Figure 1a) was conducted with the parameters U = 0.5 V, σ = 400 µΩm, and $t_{w}=40\;\mathrm{ms}$. Its temperature field is described with $\bar{T}$ = 310 °C, SD($\bar{T}$) = 196 °C, $T_{\min}$ = 82 °C, $T_{\max}$= 824 °C and marked with a thin molten and mushy zone along the faying surface; the rest of the sheet is solid. Compared to Figure 1a, the spot weld in Figure 1b exhibits lower temperature values $\bar{T}$, SD($\bar{T}$), $T_{\min}$, and $T_{\max}$ due to a reduced electrode voltage of 0.45 V. It is solid except for a mushy area at the contact layer. The Figure 1c indicates a molten and mushy phase along the faying surface, both shaped like a flat ellipse. On account of an increased voltage of 0.55 V, the temperature field of Figure 1c is overall higher than in the reference weld. An examination of the welds in Figure 1a–c shows that the temperature increases with voltage. In Figure 1d near the left-bottom corner, a slight molten pool can be observed. Furthermore, a thin mushy zone evolves along the faying surface. Compared to the reference weld, the contact resistance σ = 300 µΩm and the values of $\bar{T}$, SD($\bar{T}$), $T_{\min}$, and $T_{\max}$ are lower. An increase of the contact resistance up to σ = 500 µΩm (Figure 1e) induces higher temperatures than in the reference spot weld. As a result, an increase/decrease of the contact resistance causes higher/lower spot weld temperatures. Finally, the welding time of the reference spot weld was varied by ± 10 ms to verify the model. While Figure 1f depicts merely the onset of a fusion area, Figure 1g shows clearly an elliptically shaped weld spot. By contrast to Figure 1a, the weld temperatures in Figure 1f,g are decreased and increased, respectively. As expected, the weld spot temperature increases the longer the welding time lasts.

## 4. Discussion

### 4.1. On Computation Speed and Convergence Analysis

In Table 3, the simulation variant 4 with ∆t = 40 ms (n = 1000) exhibits a discretization error of 1.9 %, while variant 7 with ∆t = 4 µs (n = 10,000) leads to a discretization error of 0.3 %. It exemplifies the expected relation between the time step and the discretization error, i.e., the discretization error decreases with decreasing time step. Moreover, the run-time increases with shrinking time step as discussed hereinafter. The run-time for the variant 4 with ∆t = 40 µs and variant 7 with ∆t = 4 µs in Table 4 are 5 s and 9 s, respectively. In addition, the run-time relates to the discretization error conversely, i.e., the run-time increases with decreasing discretization error. By comparing variants 4 and 7 of Table 3 again, it is discernible that the run-time increases from 5 s to 9 s while the discretization error decreases from 1.9 % to 0.3 %. These relations apply to all (n,$t_{w}$)-variants in Table 3, Table 4 and Table 5 equally. From a numerical point of view, it has been shown that the relations among the time step, discretization error and run-time meet common expectations on the general behavior of finite difference methods. Another observation is that the solution converges from top to bottom for decreasing time step size. For example, in Table 5 the variants 3, 6, 9, and 12 possess mean sheet temperatures $\bar{T}$ and time steps ∆t of ($\bar{T}$ = 398 °C, ∆t = 100 µs), ($\bar{T}$ = 385 °C, ∆t = 10 µs), ($\bar{T}$= 383 °C, ∆t = 1 µs) and ($\bar{T}$ = 382, ∆t = 0.1 µs). The standard deviation SD($\bar{T}$), $T_{\min}$, and $T_{\max}$ behave analogously. However, an unexpected effect can be observed by comparing the variants 4 in Table 3, Table 4 and Table 5. The run-time increases with increasing time step ∆t for a fixed number of simulation runs. For example, the run-time amounts to 5 s, 10 s and 15 s for corresponding time steps of ∆t = 40 µs, ∆t = 50 µs and ∆t = 60 µs, although the simulation runs n = 1000 are identical in all three variants. It can be explained by the fact that an increased time step leads to higher temperature values of the intermediate simulation result. High temperature values require more computer bits of the central processor unit than lower temperature values and this coincides with higher computational costs.

The demand for fast computation models for the precise prediction of process simulation has been growing since the beginning of Industry 4.0. The idea of integrating real-time-capable digital twins into production processes to increase productivity, by reducing waste or increase quality, and use them as monitoring and control units is receiving increasing attention in industry and research. In [26] a digital twin for RSW is presented, which visualizes the temperature field. It consists of an interpolation model based on experiments and FE computations. The digital twin delivers almost identical results to the simulation model (deviation < 1%) and takes only 10 s instead of one hour (FEM model). In [27] the inherent strain and deformation method are applied to predict the total deformation of 23 resistance spot welds in a vehicle part within around 90 min. The resulting deformation, the so-called inherent deformation, is achieved by calculating the difference between the total and elastic deformation. The total deformation is identified by experiments; the elastic deformation is calculated by an FEM-tool. In [28], an equivalent parametric methodology for modelling multi-pass longitudinal welds on planar structures, such as plates and rectangular hollow sections, is introduced. The so-called welding equivalence model consists of a single and multi-layered shell, connection and beam elements and is generated by an automatic sub-program that acts on already existing FE shell model. It uses transient thermal and steady-state structural analysis to identify residual stresses and local distortions typical of multi-pass welds. Compared to classic numerical 3D models, it reduces the computing time by a factor of ten. Although the results of the studies [26,27,28] are remarkable in terms of the shortened computing time, the extended preparation time is critical; namely carrying out experiments and/or numerical simulations. It is not evident how the computational time savings compare to the additional preparation time, or whether this translates into an overall time saving. The computation speed of numerical models remains a bottleneck in the above-mentioned studies. It underlines the importance of time-efficient simulation models.

For the design of numerical programs exist at least three aspects that have a significant impact on the computing speed of transient thermal conduction problems. This includes the choice between explicit vs. implicit difference methods, iterative vs. direct solvers and structured vs. unstructured grids. Explicit methods are conditionally stable, so that a given stability criterion must be met. It limits the size of the time step to be selected and leads to long run times for simulations. Implicit methods are unconditionally stable and are not subject to this restriction. Because a larger time step may be selected for implicit methods, the simulation run time can be shorter. The disadvantage of these methods is the increased effort during implementation. Iterative solvers, such as the Gauss–Seidel process, require numerous repetitions to converge. Here, the iteration runs are canceled if the residual and/or the difference of two consecutive solution values fall below a pre-specified tolerance. Direct solvers only need one time step to reach the solution for a certain point in time. The Thomas algorithm, an example of a direct solver, represents a recursion formula that uses the boundary conditions to indicate exactly the result of the difference equation at a point in space. The prerequisite for the application of the Thomas algorithm is the presence of a linear equation system with a tridiagonal coefficient matrix. Grid-based discretization methods (FDM, FVM, FEM), distinguish between structured and unstructured grids or meshes. Unstructured meshes, which are usually used in FEM [29], have irregularly distributed nodes and their cells do not need to have a standard shape. Therefore, they are the preferred method for generating meshes in areas with complex geometries. However, the use of unstructured meshes complicates the numerical algorithm due to the inherent data management problem, which requires a special program to number and organize the nodes, edges, surfaces, and cells of the grid. In addition, linearized difference schema operators on unstructured meshes are not usually band matrices, making it difficult to use implicit schemes. The numerical algorithms based on unstructured grids are the most costly in terms of computing time and memory. Structured grids, which are the basis of the FDM, implicitly contain the order of the grid elements in their solution; the application of a program for the management of the grid elements is omitted. As a result of the ordered structure of structured grids, model equations with band matrices are created, which allow the application of time-efficient solvers. A major disadvantage of primitive FDM is that complex geometry, characterized by curves, sharp/obtuse angles, can only be modelled if losses in computational accuracy are acceptable. However, the use of elliptical grids can level out this disadvantage [30]. Regarding these three major aspects of the numerical software design, the solver algorithm was designed in favor of a short simulation run time, i.e., an implicit finite-difference method with a direct solver on a structured mesh was developed.

### 4.2. On Parameter Study

In aluminum alloy spot welds two types of nugget development have been observed in studies based on practical experiments. In one of them, melting starts as a circle around the center at the contact area. Gradually, from all sides the melting continues inwards until a complete nugget is formed [31]. In the other type initial melting is located at the center of the faying surface, before it spreads in vertical and horizontal direction outwards [13,32]. The nugget growth in the present study starts in the center and extends outwards and, thus, can be assigned to the latter type. Hereby proof for the qualitative correct simulation of the nugget development is given. Table 6 points out that the sheet temperature and the weld spot of the aluminum sheet grow with the applied electrode cap voltage, the contact resistance and welding time. These observations align with general expectations of RSW [33].

## 5. Conclusions

As mentioned at the beginning, the requirements for a model-based real-time monitoring and control system for RSW are sufficient model accuracy and computation speed. In terms of the computation speed, the simulation run-time falls below 20 s for a discretization error ≤ 1%, whereas the process cycle (squeezing-, welding-, holding-, off-phase) lasts up to a couple of seconds depending on process conditions. If the real-time simulation should run to RSW-Process simultaneously, the simulation run-time must undercut the cycle time of the spot weld process. This requires additional effort to reduce the run-time below the cycle time. In terms of software optimization, technics of high-performance computing, for example, vectorization and parallel computing as well as efficient programming possess the potential to accelerate the computation speed additionally. Moreover, hardware with more or higher processing power can support real-time simulation of RSW. After all, in combination with an adequately chosen solving algorithm the finite difference method seems to be a feasible approach for computing resistance spot welding close to real-time. Investigations to come will include the validation of the model by means of experiment.

## Figures and Tables

**Table 1:** Geometry features and dimensions.

**Geometry Feature**	**Dimension (mm)**	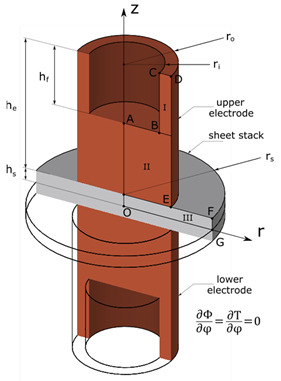
sheet radius $r_{s}$	8.8	
sheet thickness $h_{s}$	2	
inner electrode radius $r_{i}$	6	
outer electrode radius $r_{o}$	8	
electrode height $h_{e}$	23	
cooling recess $h_{f}$	10.5	
space increments Δr=Δz	0.4	
height of contact layer	$10^{-2}$	

**Table 2:** Material data set.

	Aluminum Sheet			Copper Electrode Cap
	Solid (T<660 °C)	Phase Change (T=660 °C)	Liquid (T>660 °C)	Solid
ρ (kg/$m^{3})$	2663	2385	2323	8874
$c_{p}$ (J/kg⋅°C)	1041	1194	1085	412
λ(W/(m⋅°C))	231.5	209	102	372
σ(nΩm)	56	110	270	38.7

**Table 3:** Characterization of sheet temperature field depending on (n,Δt)-variants (welding time $t_{w}=40\;\mathrm{ms}$).

**Variant**	n	Δt (μs)	$\bar{T}$ (°C)	$\mathrm{SD}(\bar{T})\;({}^{\circ}C)$	$T_{max}\;({}^{\circ}C)$	$T_{min}\;({}^{\circ}C)$	ϵ (%)	$t_{sim}\;(s)$
1	100	400	332	229	1000	79	7.4	3
2	200	200	326	218	941	82	5.5	3.5
3	400	100	320	210	900	82	3.6	4
4	1000	40	315	203	862	82	1.9	5
5	2000	20	312	200	845	82	1.0	6
6	4000	10	311	198	832	82	0.7	8
7	10,000	4	310	196	824	82	0.3	9
8	20,000	2	310	196	821	82	0.3	14
9	40,000	1	309	195	819	82	0.0	20
10	100,000	0.4	309	195	818	82	0.0	36
11	200,000	0.2	309	195	818	82	0.0	66
12	400,000	0.1	309	195	817	82	0.0	125

**Table 4:** Characterization of sheet temperature field depending on (n,Δt)-variants (welding time $t_{w}=50\;\mathrm{ms}$).

**Variant**	n	Δt (μs)	$\bar{T}$ (°C)	$\mathrm{SD}(\bar{T})\;({}^{\circ}C)$	$T_{max}\;({}^{\circ}C)$	$T_{min}\;({}^{\circ}C)$	ϵ (%)	$t_{sim}\;(s)$
1	100	500	419	302	1456	118	9.7	6
2	200	250	409	286	1368	115	7.1	8
3	500	100	398	268	1270	113	4.2	10
4	1000	50	392	260	1226	112	2.6	10
5	2000	25	388	255	1194	112	1.6	10
6	5000	10	385	250	1169	111	0.8	12
7	10,000	5	384	249	1160	111	0.5	14
8	20,000	2.5	383	248	1155	111	0.3	18
9	50,000	1	383	247	1152	111	0.3	27
10	100,000	0.5	382	247	1151	111	0.0	43
11	200,000	0.25	382	247	1150	111	0.0	73
12	500,000	0.1	382	247	1150	110	0.0	162

**Table 5:** Characterization of sheet temperature field depending on (n,Δt)-variants (welding time $t_{w}=60\;\mathrm{ms}$).

**Variant**	n	Δt (μs)	$\bar{T}$ (°C)	$\mathrm{SD}(\bar{T})\;({}^{\circ}C)$	$T_{max}\;({}^{\circ}C)$	$T_{min}\;({}^{\circ}C)$	ϵ (%)	$t_{sim}\;(s)$
1	100	600	526	408	2001	150	10.0	11
2	200	300	515	392	1918	146	7.7	13
3	600	100	501	371	1805	141	4.8	15
4	1000	60	496	364	1768	139	3.8	15
5	2000	30	490	356	1732	137	2.5	16
6	6000	10	483	348	1695	136	1.0	19
7	10,000	6	481	346	1685	136	0.6	22
8	20,000	3	480	345	1677	135	0.4	25
9	60,000	1	479	343	1671	135	0.2	37
10	100,000	0.6	479	343	1670	135	0.2	50
11	200,000	0.3	478	343	1669	135	0.0	79
12	600,000	0.1	478	343	1668	135	0.0	199

**Table 6:** simulation parameters and results of parameter study.

	U/(V)	σ/(μΩm)	$t_{w}/\left(ms\right)$	$\bar{T}$ /(°C)	$\mathrm{SD}(\bar{T}$ )/(°C)	$T_{min}/\left({}^{\circ}C\right)$	$T_{max}$ /(°C)
a	0.5	400	40	310	196	82	824
b	0.45	400	40	281	181	68	660
c	0.55	400	40	358	245	93	1094
d	0.5	300	40	291	185	71	724
e	0.5	500	40	328	209	88	901
f	0.5	400	30	254	186	44	660
g	0.5	400	50	384	249	111	1160

## Nomenclature

T	temperature	°C	r, φ, z	cylindrical coordinates	m
Q	volumetric heat source	$W/m^{3}$	t	time coordinate	s
H	latent heat	J/kg	Δz/Δr	spatial step in z/r-direction	m
L	Lorenz constant	[-]	Δt	time step	s
$c_{p}$	specific heat	J/(kg·K)	i/j	radial/axial index	[-]
Φ	electric potential	V	n	time and iteration index	[-]
α	thermal expansion factor	$K^{-1}$			
λ	thermal conductivity	W/(m⋅K)	∇	Nabla-operator	
μ	Fourier number	[-]	δ(⋅)	1st derivative difference operator	
ρ	density	kg/$m^{3}$	$\delta {\left(\cdot \right)}^{2}$	2nd derivative difference operator	
σ	specific electric resistance	μΩm			

## Appendix A

%initialization:

- geometry of sheet and electrode

- fixed material properties of electrode

- time step Δt

- number time steps n       % simulations runs

- abs error = 0.001        % first break condition

- tolerance = 0.001        % second break condition

% start electric-thermal model

for i = 1 : n

- update temperature dependent material properties

- stop 1 = 0; % initial value of first break condition

% start electric model

while (stop1 == 0 and stop2 == 0)

        - 1st sweep of Peacheman-Rachford scheme (from left to right / r-direction):

             - upper diagonalizing (1st part of Thomas algorithm)

             - backward substitution (2nd part of Thomas algorithm)

        - 2nd sweep of Peacheman-Rachford scheme (from bottom to up / z-direction)

             - upper diagonalizing (1st part of Thomas algorithm)

             - backward substitution (2nd part of Thomas algorithm)

        - calculate residuum and relative error

        - if (residuum < abs error) then stop1 = 1 else stop1 = 0 end

        - if (difference successive solutions < tolerance) then stop2 = 1 else stop1 = 0 end

        - update electric boundaries

    end

% end electric model

% start thermal model

        - 1st sweep of Peacheman-Rachford scheme (from left to right / r-direction)

             - upper diagonalizing (1st part of Thomas algorithm)

             - backward substitution (2nd part of Thomas algorithm)

        - 2nd sweep of Peacheman-Rachford scheme (from bottom to up / z-direction)

             - upper diagonalizing (1st part of Thomas algorithm)

             - backward substitution (2nd part of Thomas algorithm)

        - determine phase state of sheet (optional)

        - update thermal boundaries

% end thermal model

end

% end electric-thermal model
